# Supplementary material for: Anti-phase boundary accelerated exsolution of nanoparticles in non-stoichiometric perovskite thin films
Source: Nat Commun. 2022 Nov 5;13:6682. doi: 10.1038/s41467-022-34289-3 (PMC9637132; doi:10.1038/s41467-022-34289-3)
Supplement: Supplementary file 1 — Supplementary Information [file 41467_2022_34289_MOESM1_ESM.pdf]

Supplementary Information

Anti-phase boundary accelerated exsolution of nanoparticles in non-stoichiometric perovskite thin films

H. Han et al.

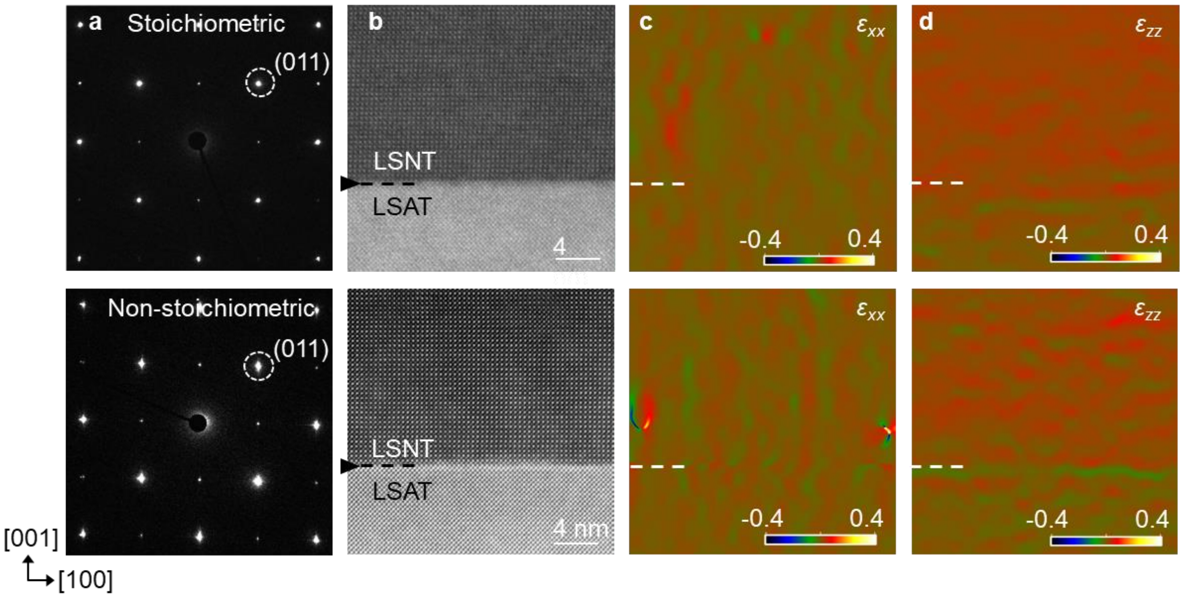

Supplementary Fig. 1. Strain analyses on LSNT thin films from TEM measurements. a, SAED patterns, **b**, STEM-HAADF images, **c**, in-plane strain (ε_{xx}) maps, and **d**, out-of-plane strain (ε_{zz}) maps for the pristine stoichiometric (top) and non-stoichiometric (bottom) films.

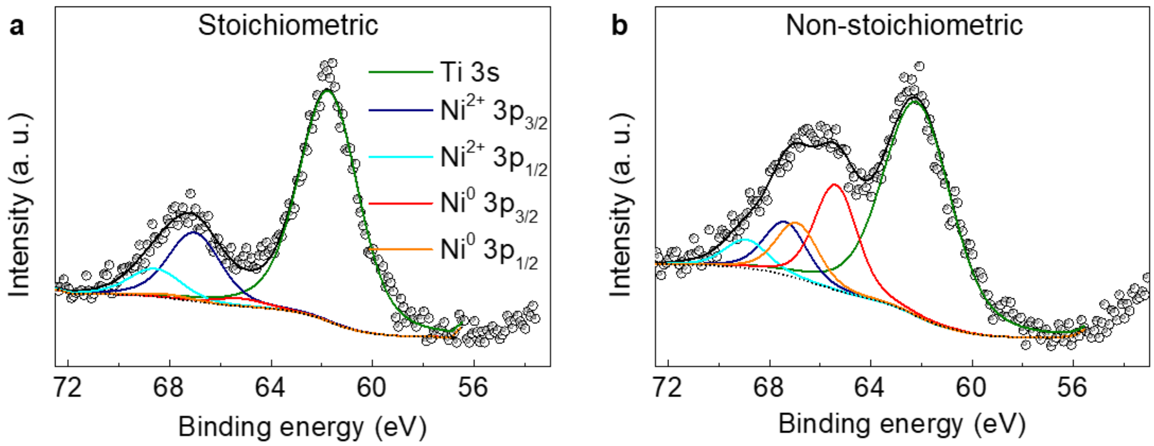

Supplementary Fig. 2. X-ray photoelectron spectroscopy (XPS) spectra of reduced thin films. XPS spectra of **a**, the stoichiometric and **b**, the non-stoichiometric thin film. The film reduction was performed in a vacuum furnace (~1×10^{-6} Torr) at 900 ˚C for 10 h.

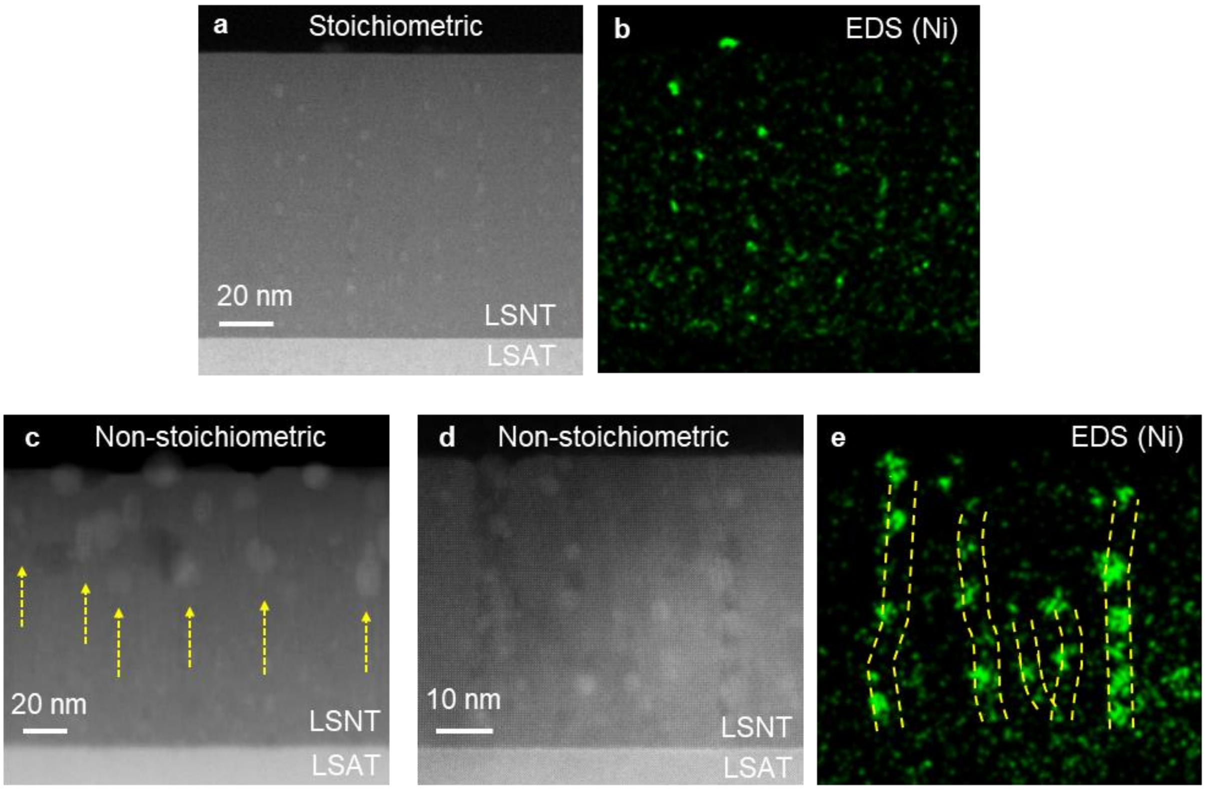

Supplementary Fig. 3. Microstructures of thin films before and after reduction. a, A cross-sectional STEM-HAADF image and **b**, the corresponding Ni EDS map of the stoichiometric 100 nm thick film after reduction in a vacuum furnace (~1×10^{-6} Torr) at 900 ˚C for 10 hrs. **c**, A STEM-HAADF image of the non-stoichiometric 100 nm thick film after reduction in a vacuum furnace (~1×10^{-6} Torr) at 900 ˚C for 10 hrs. The yellow arrows indicate traces of particle movement in the vertical direction. **d**, A cross-sectional STEM-HAADF image and **e**, the corresponding Ni EDS map of the non-stoichiometric 60 nm thick film by *in situ* reduction at 700 °C and ~10^{-7} Torr for 45 min. Ni particles tend to align vertically along the extended defects.

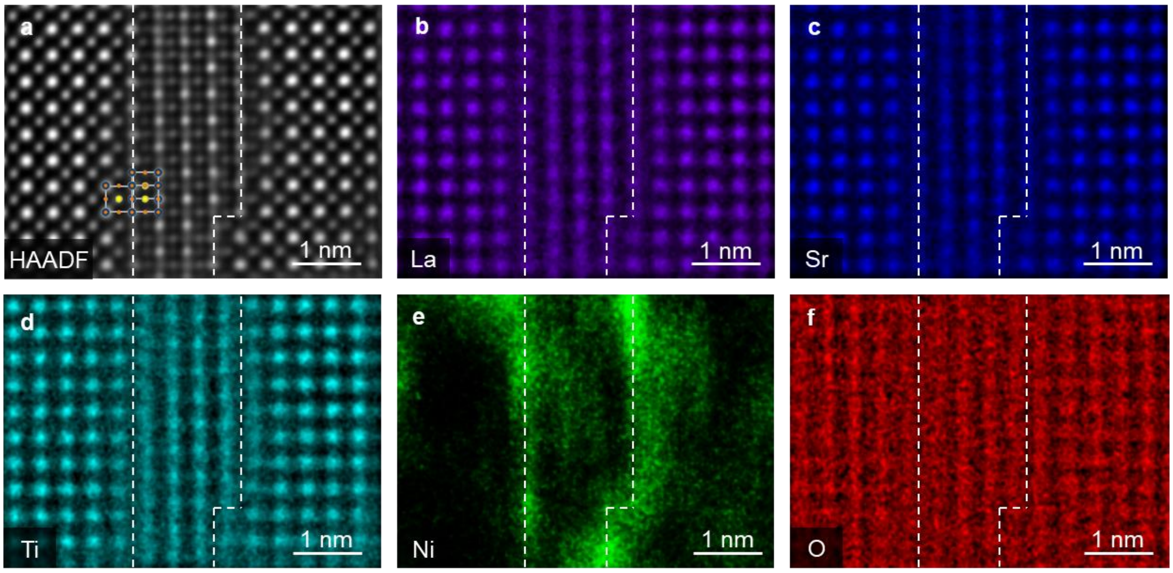

Supplementary Fig. 4. EDX elemental maps of a typical APB. a, STEM-HAADF image of APB. APB region is outlined by dashed lines. **b-c**, elemental maps of La (b), Sr (c), Ti (d), Ni (e) and O (f).

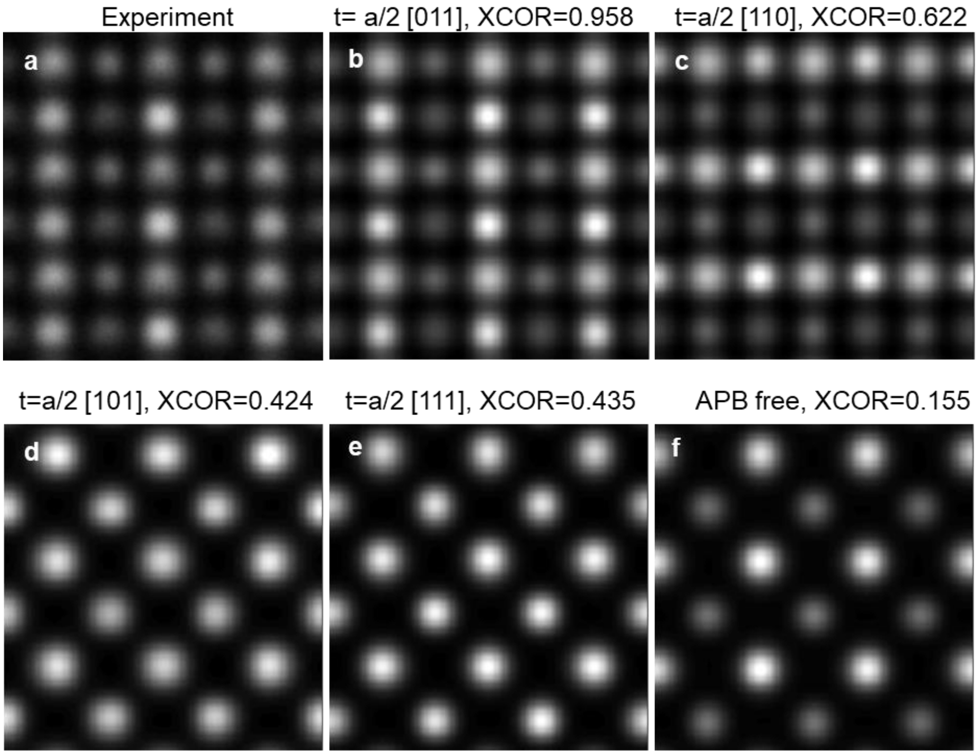

Supplementary Fig. 5. Image simulation with different translational vector of APB. a, experimental image of APB structure. **b-e**, simulated images with t=a/2 [011] (b), t=a/2[110] (c), t=a/2[101] (d) and t=a/2[111] (e). **f**, simulated image with perfect lattice. By estimating the XCOR value, t=a/2[011] (b) give the best matching result with XCOR value of 0.958.

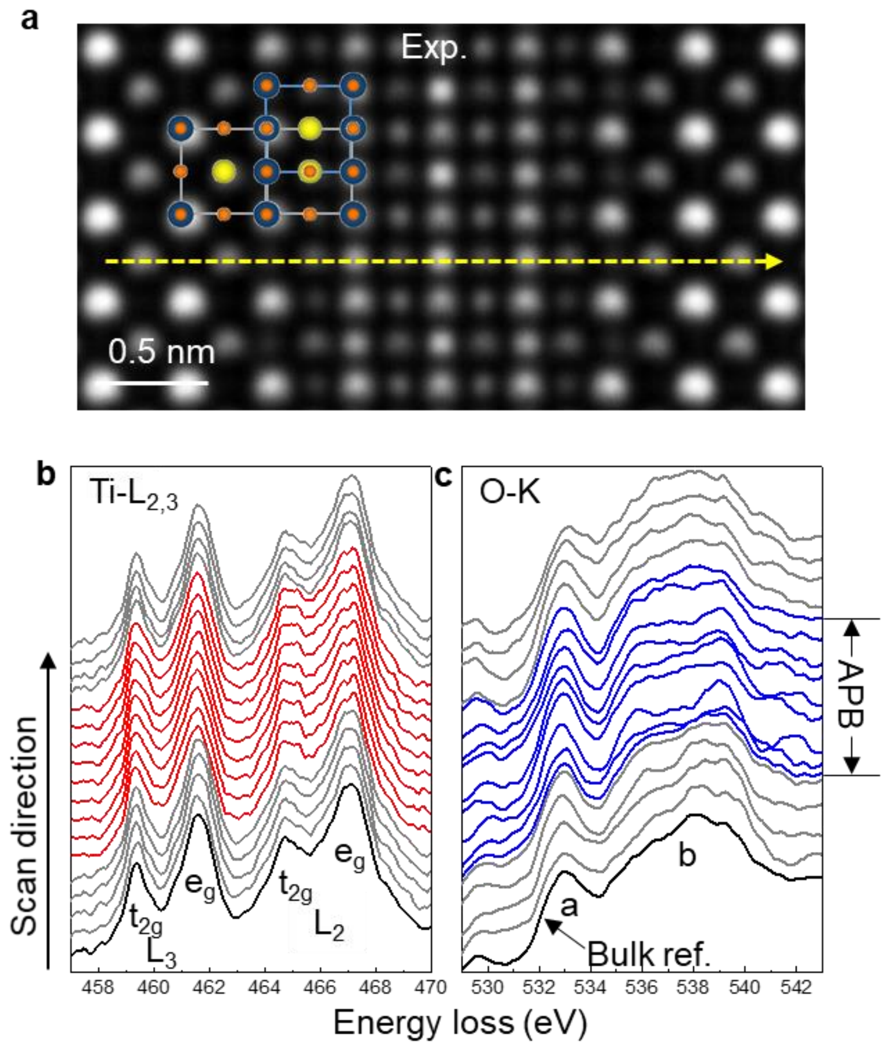

Supplementary Fig. 6. EELS analysis of APB. a, STEM-HAADF image of across APB. STEM-EEL spectra of **b,** Ti-$L_{2,3}$ edge and **c,** O-K edge along the direction of yellow dashed line marked as the arrow in (a). The red profiles in (b) and blue profiles in (c) correspond to the APB region.

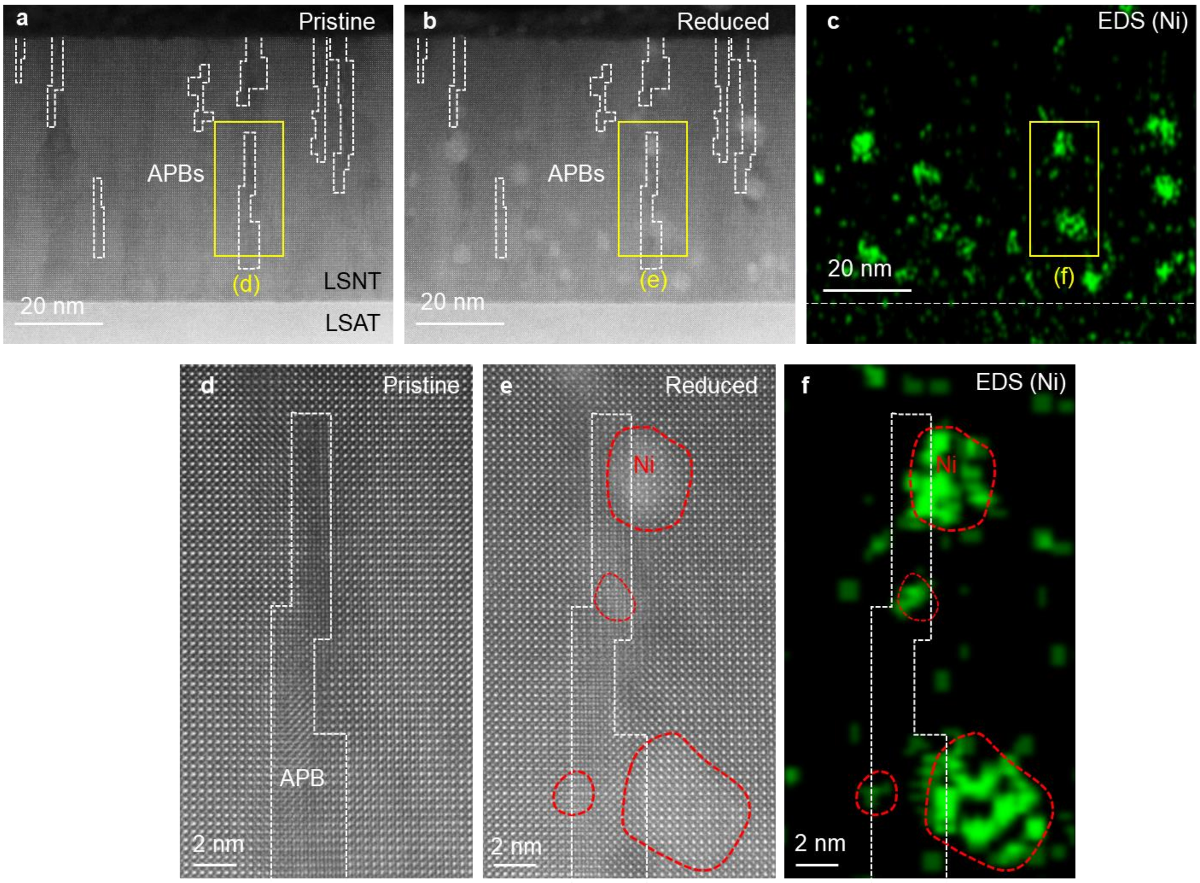

Supplementary Fig. 7. APBs-induced exsolution in the non-stoichiometric thin film. *In situ* cross-sectional STEM-HAADF images of the non-stoichiometric 60 nm thick film **a**, before and **b**, after reduction. The reduction was performed at 700 ˚C for 45 min in vacuum (~10^{-7} Torr). **c** Ni EDS map of (b). Magnified STEM-HAADF images **d**, before and **e**, after reduction and **f**, the corresponding Ni EDS maps obtained from the red rectangular region in (a), (b), and (c), respectively, revealing Ni segregation near APBs.

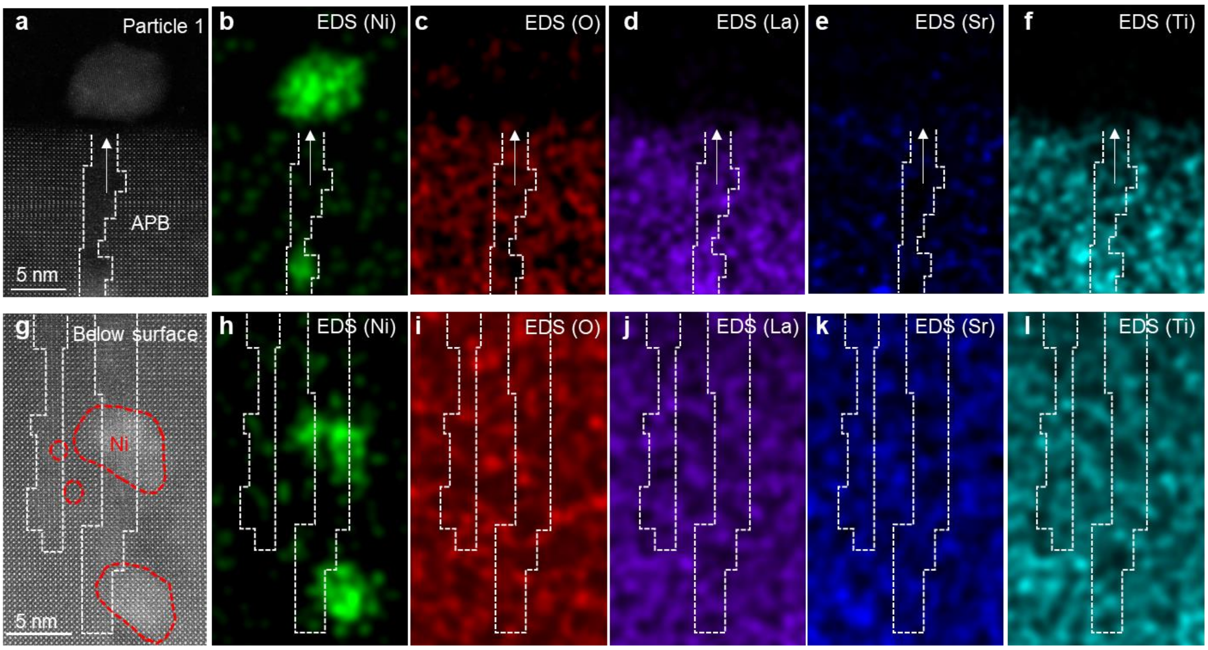

Supplementary Fig. 8. A cross-sectional STEM-HAADF images and the corresponding EDS maps of the non-stoichiometric thin film after *in situ* reduction. (a) A STEM-HAADF image of the surface area (shown in Figs. 3a and 3b) and the corresponding EDS maps of (b) Ni, (c) O, (d) La, (e) Sr, and (f) Ti. (g) A STEM-HAADF image inside the film (shown in Figs. 3d and 3e) and the corresponding EDS maps of (h) Ni, (i) O, (j) La, (k) Sr, and (l) Ti. Only Ni EDS maps correlate well with the observed particles in the STEM images. The *in situ* heating was performed at 700 °C for 45 min in vacuum (~10^{-7} Torr).

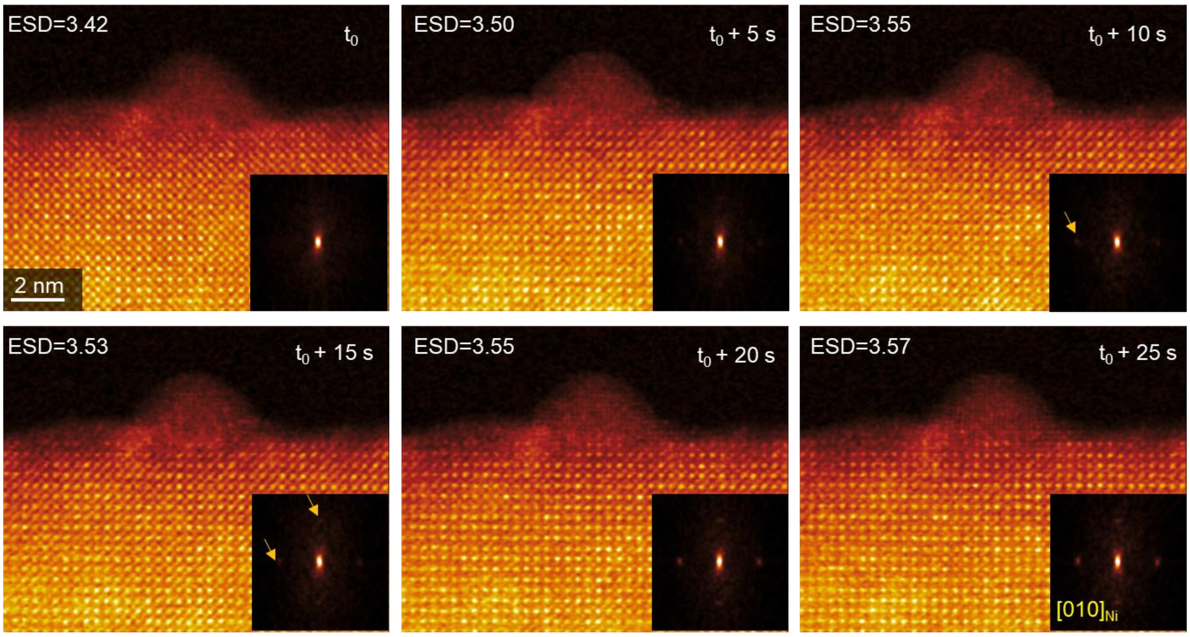

Supplementary Fig. 9. Two-step crystallization of Ni on top of LSNT surface. Time sequence STEM images showing two-step crystallization of Ni on top of LSNT surface with time span of 5 s for each frame. From t_0 to t_0+5 s, Ni at surface is growing as amorphous state. As Ni particle keep growing until the ESD value arrives around 3.55 nm at t_0+10 s, crystallization initiates with in-plane lattice appearing first as indicated in inset FFT. From t_0+15 s to t_0+25s, out-of-plane lattice gradually appears and crystalline lattice is stabilized. As a consequence, Ni is epitaxially grown on top of LSNT (001) surface with $[010]_{Ni}$ zone axis.

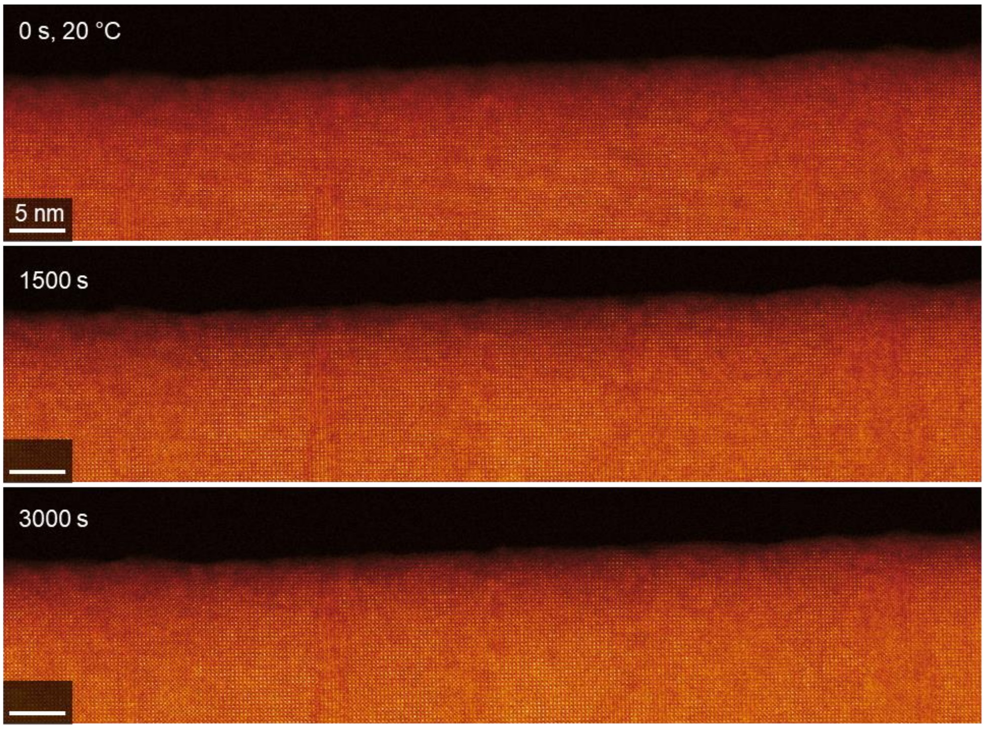

Supplementary Fig. 10. Time sequence STEM images of the pristine non-stoichiometric thin film measured at 20 ˚C. The film does not show any nucleation or crystallization when exposed to the electron beam at 20 ˚C for ~50 min. The electron beam current for *in situ* imaging the exsolution process is on the level between 10^7-10^8 e/Å^2·s under the consideration of balancing the image contrast and avoiding beam damage effect. Therefore, the observed structural fluctuations and crystallization in Fig. 5 are expected to be due to the temperature effect rather than the electron beam effect.

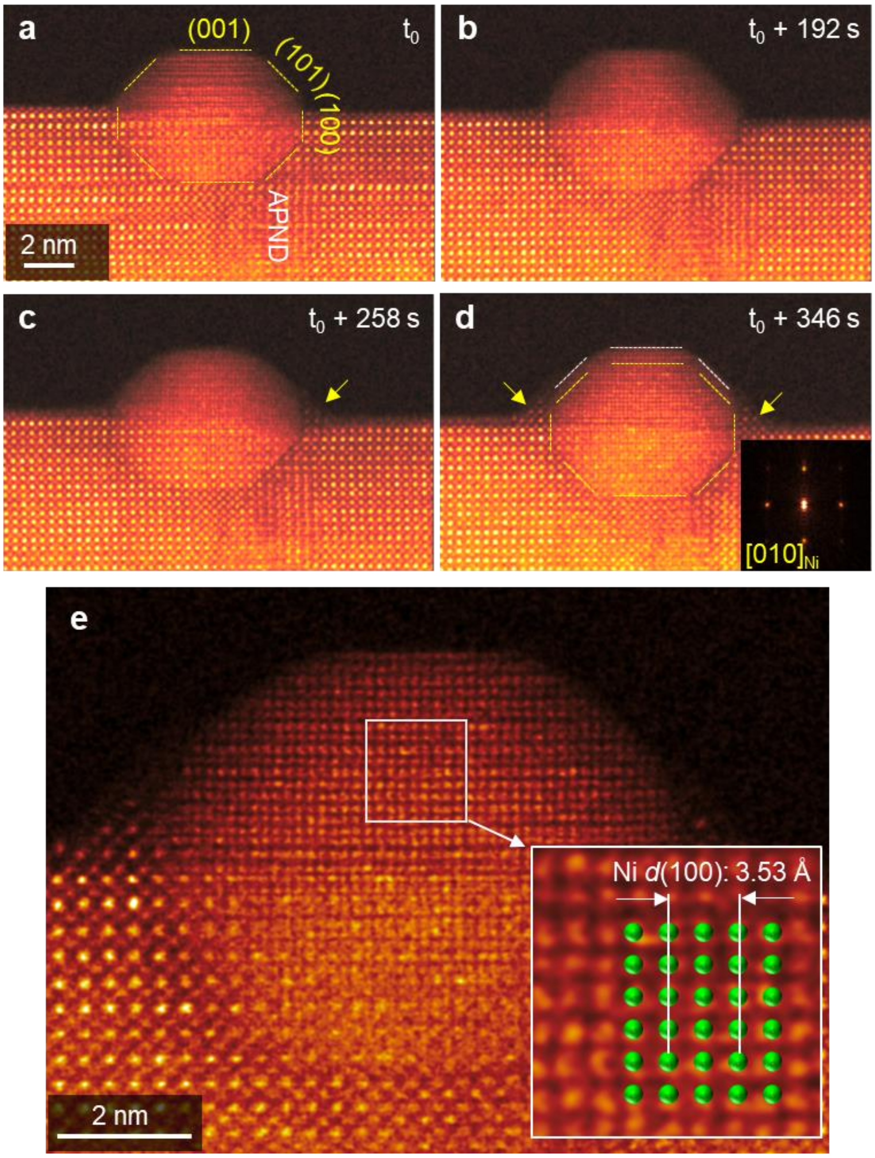

Supplementary Fig. 11. Faceted socketing of exsolved Ni particle. **a** and **b**, time sequence images showing socketed Ni particle with preferred facet epitaxially growing on top of LSNT (001) surface. The faceted surfaces are outlined by yellow dashed lines. **c** and **d**, LSNT perovskite lattice raise up and gradually socket $\{100\}_{Ni}$ surfaces of Ni particle, indicated by arrows. The size change is indicated by yellow (t_0) and white (t_0+346 s) dashed lines . No remarkable growth is recognized below the surface. **e**, A magnified STEM image of the exsolved particle on the surface, where green spheres denote Ni ions in the inset. The lattice parameter (~3.53 Å) of the particle is in good agreement with that of the Ni crystal.

Supplementary Video Captions

Supplementary Video 1. *In-situ* STEM movie showing NP formation process on top of APB, revealing the TSC mechanism. To enhance the contrast of the original movie which was recorded at 0.5 frames per second, the successive 5 frames were averaged. The measurement was done at 800 °C.

Supplementary Video 2. *In-situ* STEM movie showing faceted socketing of exsolved Ni particle. The movie is recorded at 0.5 frames per second. The measurement was done at 800 °C.
