# Supplementary material for: Anti-phase boundary accelerated exsolution of nanoparticles in non-stoichiometric perovskite thin films
Source: Nat Commun. 2022 Nov 5;13:6682. doi: 10.1038/s41467-022-34289-3 (PMC9637132; doi:10.1038/s41467-022-34289-3)
Supplement: Supplementary file 3 — Description of Additional Supplementary Files [file 41467_2022_34289_MOESM3_ESM.pdf]

Description of Additional Supplementary Files

File Name: Supplementary Movie 1
Description: In-situ STEM movie showing NP formation process on top of APB, revealing the TSC mechanism. To enhance the contrast of the original movie which was recorded at 0.5 frames per second, the successive 5 frames were averaged. The measurement was done at 800 °C.

File Name: Supplementary Movie 2
Description: In-situ STEM movie showing faceted socketing of exsolved Ni particle. The movie is recorded at 0.5 frames per second. The measurement was done at 800 °C.
